# Self-Medication Perceptions and Practice of Medical and Pharmacy Students in Serbia

**DOI:** 10.3390/ijerph19031193

**Published:** 2022-01-21

**Authors:** Ana Tomas Petrović, Nebojša Pavlović, Nebojša Stilinović, Nikola Lalović, Milica Paut Kusturica, Tihomir Dugandžija, Dragana Zaklan, Olga Horvat

**Affiliations:** 1 Department of Pharmacology, Toxicology and Clinical Pharmacology, Faculty of Medicine, University of Novi Sad, 21000 Novi Sad, Serbia; nebojsa.stilinovic@mf.uns.ac.rs (N.S.); milica.paut-kusturica@mf.uns.ac.rs (M.P.K.); olga.horvat@mf.uns.ac.rs (O.H.); 2 Department of Pharmacy, Faculty of Medicine, University of Novi Sad, 21000 Novi Sad, Serbia; nebojsa.pavlovic@mf.uns.ac.rs (N.P.); dragana.zaklan@mf.uns.ac.rs (D.Z.); 3 Psychiatry Clinic, Clinical Center of Serbia, 11000 Belgrade, Serbia; nikolalovic@hotmail.com; 4 Department of Epidemiology, Faculty of Medicine, University of Novi Sad, 21000 Novi Sad, Serbia; tihomir.dugandzija@mf.uns.ac.rs

**Keywords:** self-medication, students, medical knowledge, Serbia, herbal drugs, pharmacy

## Abstract

Background. Attitudes towards conventional and complementary medicine among future healthcare professionals can impact their future pharmacotherapy practice. This study aimed to determine the prevalence and predisposing factors related to self-medication among medical and pharmacy students. Methods. This cross-sectional questionnaire-based study was performed at the Faculty of Medicine, University of Novi Sad, Serbia, on first- and final-year students of medicine and pharmacy. The multivariate Poisson regression model with robust variance was used to identify the main predictors of self-medication. Results. The overall self-medication prevalence in the past year was 81.3%. Independent risk factors for self-medication identified in the regression analysis were the final study year, housing condition, i.e., living in a leased apartment or in a student dormitory in comparison to living with parents, and cigarette consumption. The conventional drugs were the most frequently used, mostly for the symptoms of cold and pain. Final-year students had more confidence in conventional medicines than in herbal drugs and were more aware of the risks of their concomitant use. Conclusion. Self-medication is highly prevalent among students of medical sciences, especially among final-year students. Increased medical knowledge led to the higher awareness of the drug interaction risks.

## 1. Introduction

Self-medication is a global phenomenon that has been receiving considerable attention within the healthcare systems worldwide [1]. According to the World Health Organization (WHO), self-medication is defined as one element of self-care and represents the selection and use of medicines by individuals to treat self-recognized illnesses or symptoms [2].

Increased availability of drug and disease information, especially on the Internet, together with increased interest in one’s health, has resulted in the higher demand for more direct involvement of patients in the decision-making [3]. An increase in self-medication practice has led to the large number of people today using over-the-counter (OTC) drugs for minor ailments such as headache, cold, flu, muscle pain, etc., without consulting a medical professional [4]. Furthermore, patients often rely on herbal, traditional and homeopathic preparations, which are generally perceived as being more affordable, natural and safer than conventional drugs [5]. However, this is not necessarily true as all effective drugs may produce adverse reactions, and herbal medicines are no exception [6]. Besides, many herbal medicines are complex mixtures of more than one active ingredient, which increases the possibility of interactions with conventional drugs when used concomitantly [7].

Responsible self-medication involves the use of medicines that are approved, available without prescriptions (OTC drugs) and considered relatively safe and effective when used as directed [8]. The term ‘responsible self-medication’ has been introduced to emphasize potential individual and social benefits linked to self-medication practices, including increased access to effective treatment, reduced pressure on the healthcare system due to a decreased number of visits to physicians, increased patient autonomy and reduced costs to third-party payers, such as government or insurance companies [9]. On the contrary, irresponsible or irrational self-medication, usually with prescription-only medicines, brings a higher risk of direct harm to the patient in the form of adverse drug reactions [10]. However, even when used responsibly, self-medication is linked to several risks for self-medicated individuals and the community, such as a delay in diagnosis and incorrect diagnosis, the occurrence of drug-induced diseases and the growth of public health expenditure [11]. Although the WHO recognizes the importance of self-medication in the healthcare system, it points out that responsible self-medication requires adequate support of medicinal products with all the necessary information such as a patient information leaflet (PIL) and improvements in people’s general knowledge, level of education and socio-economic status [12].

The prevalence rates of self-medication in the general public were shown to be high all over the world. In a systematic review involving 70 studies on self-medication practices, the reported prevalence of self-medication was higher than 50% in 53.8% of the studies. It was also demonstrated that self-medication was more prevalent in developing countries [13]. The prevalence rates are continuously rising, particularly among the youth, which can be attributed to socio-economic factors, lifestyle, greater availability of medicinal products, easy access to health information, etc. [14].

The self-medication practice in Serbia was assessed in the general population, and self-medication with prescription drugs appeared to be a rather common practice, which conflicts with the concept of responsible self-medication. According to the collected data from the households in Novi Sad, Serbia, more than half of the listed drugs were purchased at pharmacies without a prescription. The cases of irresponsible and inadequate drug use, such as their use for the wrong indication, incorrect dosage and insufficient therapy duration, were also identified among Serbian respondents [15].

Self-medication was found to be more common among physicians and medical students (above 50% in 76% of studies) in comparison to the general public, and inappropriate self-treatment has been identified as an issue since many physicians and medical students find it challenging to be a patient as a result of their medical training and attitudes [16]. Therefore, the results of previous studies and suggested interventions for the general population cannot be extrapolated to the population of students in medical sciences, since they have greater knowledge of diseases and therapies and better access to healthcare information as well [17]. It was demonstrated that the study program, as well as the years of education, can make certain students prone to self-medication. The results of the survey conducted in Slovenia demonstrated that more than 90% of university students reported the use of some sort of self-medication during the previous year, with a higher prevalence among healthcare students in comparison to non-healthcare students and among last-year students in comparison to the first-year students. However, healthcare-related education in students and young adults was shown to lead to more responsible self-medication [18].

In Serbia, self-medication prevalence among medical students at the University of Belgrade was reported to be around 80% in 2012 [19]. There are no available data on the frequency of self-medication and its risk factors among students in the Autonomous Province of Vojvodina, but the results of recent research among the general population have identified self-medication as an issue that deserves additional consideration [3,15,20].

Self-medication has not been investigated among students in Serbia in the last eight years and never in the Autonomous Province of Vojvodina, which has a different population pattern in comparison to central Serbia. Vojvodina is ethnically one of the most heterogeneous regions of Europe with specific cultural, ethnic, educational and social characteristics of the population [21]. The aim of our study was to determine the prevalence, attitudes and factors associated with the practice of self-medication among first- and last-year medicine and pharmacy students at the University of Novi Sad, Serbia. To the best of our knowledge, this is the first comparative analysis between medical and pharmacy students in Serbia regarding self-medication practices.

## 2. Materials and Methods

### 2.1. Study Design and Setting

The survey was conducted on a sample of first- and final-year students of integrated academic studies of medicine and pharmacy at the Faculty of Medicine, University of Novi Sad, Serbia, between 15 December 2017 and 15 January 2018.

The sample size, as calculated according to the prevalence of self-medication of 86.2% (based on average of prevalence rates observed in previous studies in Serbia, Slovenia and Romania) [18,19,22] with a confidence level of 0.95 and precision level of 0.05,was 183. Sample size necessary for the comparison between two groups (medicine vs. pharmacy or first-year vs. final-year) assuming the prevalence of self-medication of 92.8% and 79.9% [19,21] with a confidence level of 0.95 and precision level of 0.05 was 192.

The questionnaire (Supplementary File S1) was created in an electronic version using the Google forms platform and it was distributed to the students of selected study years via their official e-mail addresses obtained during enrollment in the first year of the studies. In Serbia, studies are organized as integrated master studies with pharmacy being a 5-year program and medicine a 6-year program. All registered students of the 1st year of medicine and pharmacy program and 5th year pharmacy and 6th year medical students were eligible to participate in the study. There were 221 medical and 88 pharmacy students enrolled in the 1st year, and 165 medical and 81 pharmacy students enrolled in the final year of the studies. Therefore, a questionnaire was sent out to 564 e-mail addresses, and 192 students completed the questionnaire. Detailed information about the survey was provided on the first page of the questionnaire. Before each questionnaire was filled in, all subjects gave informed consent. The obtained data were retrieved in .csv format, imported in Excel 2016 and further statistically analyzed.

### 2.2. The Questionnaire

The questionnaire was created for the purpose of this research by a combination of questions from the previously performed surveys [18,19,23,24,25], with modifications necessary to ensure correct answers to questions and claims and to be more suitable for prospective pharmacists and medical doctors who will be able to prescribe and dispense medication in the future. The content, comprehension, readability and design of the questionnaire were pre-tested on 30 students. The questionnaire contained two parts. The first part consisted of 13 questions. The first question was close-ended and referred to the self-medication practice in the previous 12 months. For students who answered yes on the first question, three additional multiple response questions (multiple answers allowed) examined the reasons for self-medication, indications and groups of drugs used in self-medication practice. These questions were followed by two questions (multiple answers allowed) about the sources of medicines used for self-medication and sources of information. Eventually, the last six questions examined students’ self-medication practices with herbal, traditional and homeopathic remedies and their views on concomitant use with conventional drugs. The second part of the questionnaire (10 questions) referred to socio-demographic characteristics of the students and life habits.

### 2.3. Data Analysis

Statistical analysis was performed with IBM SPSS software (SPSS 22.0 for Windows, SPSS Inc., Chicago, IL, USA). Out of descriptive statistical methods, measures of central tendency (arithmetic mean), measures of variability (standard deviation) and frequency were used. Chi-square test was used to test the differences between nominal data (frequencies), while *t*-test for independent samples and ANOVA with Tuckey post hoc test were used for numerical variables. Man–Whitney and Kruskal–Wallis were used for numeric data with non-parametric distribution. In order to analyze the predisposing and associated factors related to self-medication, a multivariate Poisson regression model with robust variance was used [26,27]. The dependent variable was the practice of self-medication in the last 12 months (yes or no). The independent variables tested were: gender (female/male), study program (pharmacy/medicine), study year (final/first), housing conditions (living with parents/leased apartment or room/student dormitory), cigarette consumption (yes/no), regular alcohol consumption (yes/no), chosen general practitioner (yes/no), having a chronic illness (yes/no) and taking medication for chronic illnesses (yes/no). The association of respondents’ characteristics with self-medication practice was first evaluated using univariate models. A multivariate Poisson regression model with robust variance included those predictors which were statistically significant in the univariate models, at a significance level of 0.05. Alcohol consumption as an independent variable was not included in the multivariate model due to its multicollinearity with cigarette consumption. Results were reported as prevalence ratios (PRs) with 95% confidence intervals (CIs). All *p*-values less than 0.05 were considered significant.

## 3. Results

Out of 564 questionnaires distributed by e-mail to the medicine and pharmacy students, the survey was completed by 192 students (response rate 34.0%). A total of 109 medical students (67 in first year, 41 in final year, 1 without answer) and 83 students of pharmacy (41 in first year, 36 in final year, 6 without answer) participated in the survey. Given that seven students did not answer the question regarding the study year, a comparison based on the self-medication practices between students of different study years was performed on 185 students. The majority of respondents were female (84.5%), and there were no statistically significant differences in the proportional share of studied student groups in the sample (medicine vs. pharmacy, first year vs. final year). The average age of students in the medicine program was 19.23 ± 0.53 (18–21) and 23.63 ± 1.02 (22–28) for the first and the sixth year, respectively. For pharmacy students, the average age was 19.07 ± 0.47 (18–20) and 23.32 ± 0.84 (22–26) for the first and the fifth year, respectively. Basic demographic characteristics of the respondents and data on their living habits are shown in Table 1.

**Figure 1:**
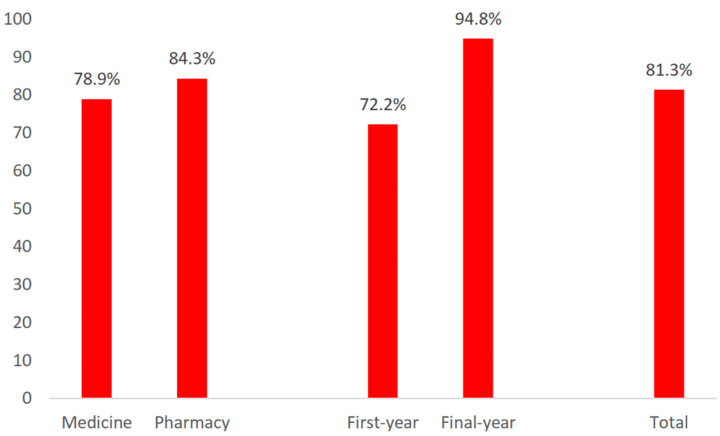
Self-medication prevalence among first- and last-year medicine and pharmacy students.

Self-medication in the previous 12 months was reported among 156 (81.3%) respondents. A statistically significantly higher number of students in the final year of their studies decided to self-medicate compared to students in the first year (*p* < 0.001), while the frequency of self-medication practice between medicine and pharmacy students did not differ significantly (78.9% vs. 84.3%) (Figure 1).

The responses of the first- and final-year pharmacy and medicine students to the question of the main reasons for their self-medication behavior in the previous year are shown in Table 2. The most common reason why students did not self-medicate was the fact that they did not have a need for self-medication (70.3%), while fewer students were concerned about the safety of taking drugs self-initiatively (29.7%). Out of 156 students who reported self-medication, the majority indicated mild symptoms that did not require a visit to the doctor as the main reason for that practice (69.2%), followed by a previous positive experience with the same medicine (42.3%).

**Figure 2:**
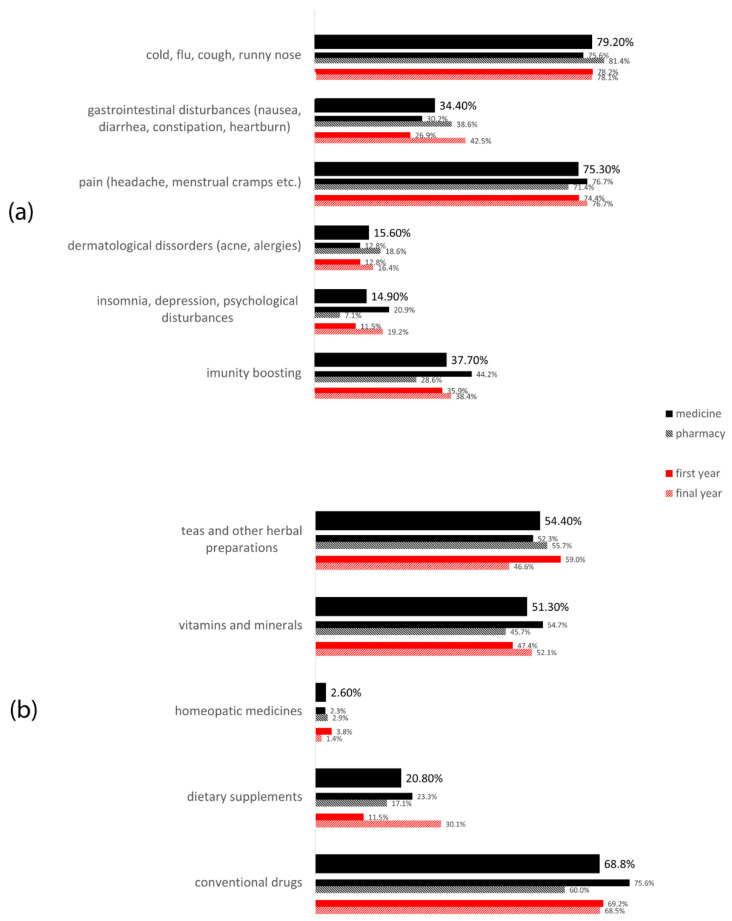
Identified indications (**a**) and types of drugs (**b**) used for self-medication.

Figure 2 shows the most frequent indications and drug types used for self-medication among the respondents. The most common symptoms and diseases that students self-treated included cold and flu (cough, nasal discharge, etc.) and pain (headache, back pain, menstrual pains, etc.) with frequencies above 75%. About 38% of students were taking medication on their own in order to improve their general health and wellbeing and to boost immunity. Besides, more than a third of surveyed students reported gastrointestinal disturbances (constipation, diarrhea, nausea, vomiting, etc.) as a reason to use self-medication. A higher number of medicine students who reported self-medication used medicines for insomnia, depression and other psychological disturbances, as well as for immunity boosting, in comparison with pharmacy students (20.9% vs. 7.1% and 44.2% vs. 28.6%, respectively; *p* = 0.036). Regarding the drug types used for self-medication, it can be observed that the majority of students in our research decided to use conventional drugs (68.8%), although more than half of respondents used vitamins and minerals, as well as teas and other herbal preparations.

The most common way of obtaining medicines for self-medication was through pharmacies (91.7%), although almost half of respondents (44.2%) reported the use of medicines remaining from earlier treatments stored in home pharmacies as a source of drugs for self-medication. No statistically significant differences were found between study programs or study years regarding the sources of medicines for self-medication. The main source of information that students relied on when choosing medicines for self-medication was a physician’s advice from previous visits regarding a disease that presents with similar symptoms (51%). Besides, more than a third of surveyed students selected a medication based on pharmacists’ advice in the pharmacy and on information from professional literature, such as class books (Table 3). A statistically significantly higher number of final year students relied on professional literature in comparison to the first-year students (*p* < 0.001).

The prevalence of self-medication practice with herbal medicines in combination with conventional drugs and attitudes of the first- and last-year medicine and pharmacy students towards their efficacy and safety are presented in Table 4. A total of 38% of respondents reported simultaneous use of conventional and herbal medication, which represents the significant majority of students who self-medicated with herbal drugs (54.4%). A slightly higher number of medical students and students in their final study year decided to use herbal and conventional drugs concomitantly, in comparison to pharmacy students and students in their first year, respectively. However, these differences between different study programs and study years were not statistically significant (*p* = 0.258, *p* = 0.304). The most common explanations for simultaneous use of these medicines reported by all investigated student groups were their beliefs in the safety of this practice and the fact that conventional and herbal drugs were not used for the same indication. Medicine students believed at a significantly higher rate that concomitant use of herbal and conventional drugs cannot be harmful in comparison to pharmacy students (68.2% vs. 44.8%, *p* = 0.035). The majority of last-year medicine and pharmacy students (80.5%) would continue their therapy with conventional medicine in the hypothetical situation where they are required to choose between these two options for the same indication, which is statistically significantly higher in comparison to the first-year students (47.2%, *p* = 0.045). A statistically significantly higher percentage of students in their finishing years of medicine and pharmacy also claimed that standard drugs are more effective than herbal remedies when compared to the first-year students (*p* = 0.01).

There was a statistically significant difference in the share of pharmacy students in comparison to medical students (*p* = 0.001) who believed that conventional medicines have less adverse effects than herbal drugs (20.7% vs. 13.6%). A larger share of first-year students (37.6%) in comparison to final-year students (28.9%) thought that herbal drugs have less adverse effects than conventional drugs (*p* = 0.004).

Eventually, 47.7% of respondents who reported concomitant use of herbal and conventional medicine and who obtained them at a pharmacy (*n* = 46) claimed that they were warned by a pharmacist about the risks of this practice.

The results of the multivariate Poisson regression model with self-medication as a dependent variable are presented in Table 5. The model contained nine predictors that were compared in a sample of 192 respondents with 156 of them having the outcome of interest (reported self-medication in the previous 12 months). In the multivariate Poisson regression model with robust variance, the characteristics of respondents associated with self-medication practice were shown to be: (1) the final study year (B = 0.246; *p* < 0.001), (2) housing conditions, i.e., living in a leased apartment/room (B = 0.220; *p* = 0.008) and in a student dormitory (B = 0.228; *p* = 0.006) in comparison to living with parents as a reference category and (3) cigarette consumption (B = 0.180; *p* = 0.001).

Self-medication prevalence in final-year students was 28% higher in comparison to the first-year students (95% vs. 72%, PR: 1.28, 95% CI: 1.13–1.44). Furthermore, self-medication prevalence in students living in a leased apartment (87%, PR: 1.25, 95% CI: 1.06–1.47) and in a student dormitory (90%, PR: 1.33, 95% CI: 1.09–1.64) was higher than in students living with parents, as a reference category (71%). Finally, smokers were shown to have higher self-medication prevalence when compared to the group of students that did not consume cigarettes (97% vs. 78%, PR: 1.20, 95% CI: 1.08–1.33).

## 4. Discussion

Considering self-medication as a significant issue in the general population and among health science students as well [16], we aimed to determine the prevalence, attitudes and risk factors of self-medication in a sample of the first- and final-year medicine and pharmacy students at the University of Novi Sad, Serbia.

According to the results of this research, over 80% of medicine and pharmacy students self-medicated in the previous 12 months. The frequency of self-medication practice reported in our study is in accordance with the results of the research performed on a sample of 1296 medical students at the University of Belgrade, Serbia, which indicated a self-medication prevalence of about 80% as well [19]. In a recent large multinational study among pharmacy students in four European countries, the prevalence rates of self-medication were shown to be 69.6% in the Czech Republic, 71.5% in Italy, 85.9% in Romania and 93.9% in Spain [22].

In our study, final-year medicine and pharmacy students opted for self-treatment at a significantly higher percentage (94.8%) compared to the first-year students (72.2%), while there was no statistically significant difference between the students of different study programs in terms of self-medication prevalence. The study conducted at the University of Applied Health Studies in Zagreb, Croatia, investigated the attitudes about self-medication for pain relief and features of self-medication in first-year students, and no differences between the six study programs were determined [28]. Similarly, self-medication prevalence was found to be similar in medicine and pharmacy students in Bangladesh, although pharmacy students were more aware of self-medication risks than medicine students [29]. In a study conducted in Jordan, pharmacy students practiced self-medication at a higher rate than medicine students (82.9% vs. 74.4%) [30].

According to the results of multivariate Poisson regression, the main predictors of self-medication were the study year, housing conditions and cigarette consumption. The higher self-medication prevalence among final-year students in our study is in accordance with the research from the region since self-medication was found to be more common in the final-year students at the University of Ljubljana, Slovenia [18]. Similarly, age has been identified as an independent risk factor for self-medication among medical students in Belgrade, Serbia [19]. Studies conducted in other countries (Turkey, Jordan, Bosnia and Herzegovina and the United Arab Emirates) have also demonstrated the correlation between the frequency of self-medication and the study year [25,30,31,32]. This change in the pattern of self-medication practice is probably due to the fact that along with the increase in knowledge, students’ confidence in their ability to identify the illness and choose the right therapy without consulting a physician grows as well.

Poisson regression has shown that students living independently from their parents, in a rented apartment or in a student dormitory, had significantly higher self-medication prevalence than those who live with their parents. On the contrary, the results of the study from the Middle East showed that medical students and interns living with parents were more prone to self-medication practice with analgesics [31]. However, due to cultural differences, the results are not directly comparable.

Students who consume cigarettes had a 20% higher self-medication prevalence than non-smokers in our study. In a survey among the students at the University of Belgrade, the consumption of alcohol was related to a 50% higher prevalence of self-medication [19]. Furthermore, illicit drug use as a lifestyle habit was identified as a risk factor for self-medication among university students in Brazil [33]. In our study, alcohol consumption was identified as a self-medication predictor in univariate models as well. However, this independent variable was not included in the multivariate model due to its multicollinearity with cigarette consumption.

The main reasons for self-medication practice in our study included the perception that the health issue is not serious and does not require going to a doctor (69.2%), as well as having a previous positive experience with the same medicine (42.3%). Given that many diseases have the same or similar symptoms, high self-confidence and a positive attitude to self-medication among students might lead to a higher risk of a wrong diagnosis and inappropriate therapy. Similarly, the most common reason for self-medication among medical students in Sarajevo, Bosnia and Herzegovina, was previous experience with the same drug and the belief they could successfully treat themselves [34]. The reasons against self-medication in our study were in agreement with the results obtained for students in other countries since around 30% of respondents in our study reported the risk of adverse effects and the wrong therapy as reasons deterring them from practicing self-medication [35,36].

The majority of investigated students decided to use medicines on their own for treating symptoms of cold and flu (79.2%) and for relieving pain (75.3%), while about 38% of respondents used medicines self-initiatively in order to improve the general state of the body and immunity. These results are in agreement with the results of research on self-medication in the general population in Novi Sad, where pain and respiratory infections were also stated as the most frequent causes of self-medication [15].

The largest number of students (92%) purchased the needed medicine from their community pharmacy. It is noteworthy that home pharmacies and leftover medicines from previous treatments were an important source in self-medication practice (44.5%). For some drug classes, leftover medicines can be a sign of poor medication adherence in patients, as recently found in the general public of Novi Sad, Serbia [3,20].

According to the results of our research, a total of 38% of respondents reported simultaneous use of conventional and herbal medication, which represents the significant majority of students who self-medicated with herbal drugs. The prevalence of self-medication with herbal preparations among medicine and pharmacy students in Novi Sad (54.4%) was higher in comparison to the prevalence among students of medical sciences in Palestine (39.7%). It should be noted that the same study demonstrated that non-medical university students were significantly less inclined to use herbal remedies in self-therapy practices (31.5%) [36]. Furthermore, the use of herbal/homeopathic remedies in the previous 12 months was reported among 27.7% of high-school students in the United Arab Emirates [37].

In our study, about 71.1% of the first-year students who used herbal and conventional drugs concomitantly believed that this practice is not harmful but only useful, which may be explained by their limited medical knowledge at the start of their studies. This can lead to inappropriate self-treatment and exposure to all risks related to inadequate use of drugs. The majority of final-year students (about 81%) would continue their therapy with conventional medicine if required to choose one option, as opposed to first-year students (about 47%). A significantly higher number of students in their final year in comparison to those in their first year considered conventional drugs to be more effective and safer than herbal remedies, which can be explained by the fact that final-year students, during their education, gain knowledge on the functioning of herbal remedies and the possible interactions between herbal and standard medicines that should not be ignored.

In the present study, almost half of the respondents who obtained herbal and conventional medicine at a pharmacy and used them concomitantly received advice from a pharmacist. These results indicate there is still room for improvement in terms of the important role of a community pharmacist in providing herbal remedies for self-medication, which has been identified in previous studies as well [24,25]. In a study conducted among Spanish pharmacy students, it was shown that there was a significantly higher percentage of respondents who self-medicated based on the advice of friends or family than those who consulted a pharmacist before taking a drug [22].

Some weaknesses of our study need to be noted. Firstly, this is a cross-sectional study with the limitation of collecting data on all variables only at a single point in time. Secondly, information bias cannot be excluded since the survey is based on self-reported data regarding activities from the previous 12 months, and it is possible that some incorrect data were given due to forgetfulness (recall bias). In addition, we did not ask about the exact active agents and drug classes used for self-medication, and we were not able to differentiate between legal and illegal self-medication practices. Moreover, due to ethical issues, we did not ask students in detail about their demographic characteristics such as religion or income level, which may have led to certain confounding factors being omitted. Eventually, the survey was performed at only one institution in the country. However, this survey included a representative sample of medicine and pharmacy students at the beginning and the end of their studies, and these results may serve as an excellent starting point for further investigation on self-medication practice as an emerging public health issue.

## 5. Conclusions

Self-medication was found to be a common practice among students of medical sciences at the University of Novi Sad, Serbia. The study program (medicine vs. pharmacy) students were enrolled in did not have a major influence on the rate of practicing self-medication. However, students in the final years of their studies were much more likely to self-medicate. This might be the consequence of healthcare-related education, which has led to greater confidence in practicing self-medication. The attitudes and behavior of future health professionals regarding self-medication can impact their future practice. Assessing how responsible or irresponsible self-medication practices among students of medical sciences in our setting are remains to be addressed, which warrants further studies.

## Figures and Tables

**Table 1:** Socio-demographic profile and living habits of respondents (*n* = 192).

Characteristics	Study Program		Study Year			Total *n* (%)
	Medicine *n* (%)	Pharmacy *n* (%)	First *n* (%)	Final *n* (%)	No Answer *n* (%)	
Gender						
Female	91 (83.5%)	73 (87.9%)	91 (84.3%)	68 (88.3%)	5 (71.4%)	164 (84.5%)
Male	18 (16.5%)	10 (12.0%)	17 (15.7%)	9 (11.7%)	2 (28.6%)	28 (14.4%)
Housing conditions						
Living with parents	42 (38.5%)	27 (32.5%)	37 (34.3%)	29 (37.7%)	3 (42.9%)	69 (35.9%)
In a student dormitory	56 (51.4%)	45 (54.2%)	57 (52.8%)	40 (52.0%)	4 (57.1%)	101 (52.6%)
In a leased apartment	10 (9.2%)	11 (13.3%)	13 (12.0%)	8 (10.4%)	0 (0.0%)	21 (10.9%)
No answer	1 (0.9%)	0 (0.0%)	1 (0.9%)	0 (0.0%)	0 (0.0%)	1 (0.5%)
Cigarette consumption						
Yes	17 (15.6%)	17 (20.5%)	13 (12.0%)	19 (24.7%)	2 (28.6%)	34 (17.7%)
No	92 (84.4%)	66 (79.5%)	95 (88.0%)	58 (75.3%)	5 (71.4%)	158 (82.3%)
Alcohol consumption						
Yes	4 (3.7%)	12 (14.5%)	3 (2.8%)	13 (16.9%)	0 (0.0%)	16 (8.3%)
No	105 (96.3%)	71 (85.5%)	105 (97.2%)	64 (83.1%)	7 (100.0%)	176 (90.7%)
Chosen general practitioner						
Yes	87 (79.8%)	60 (72.3%)	86 (79.6%)	58 (75.3%)	3 (42.9%)	147 (76.6%)
No	22 (20.2%)	23(27.7%)	22 (20.4%)	19 (24.7%)	4 (57.1%)	45 (23.4%)
Chronic illness						
Yes	11 (10.1%)	8 (9.6%)	10 (9.3%)	8 (10.4%)	1 (14.3%)	19 (9.9%)
No	98 (89.9%)	75 (90.4%)	98 (90.7%)	69 (89.6%)	6 (85.7%)	173 (90.1%)
Taking medication for chronic illnesses						
Yes	7 (6.4%)	6 (7.2%)	4 (3.7%)	8 (10.4%)	1 (14.3%)	13 (6.8%)
No	101 (92.7%)	71 (85.6%)	101 (93.5%)	66 (85.7%)	5 (71.4%)	172 (89.6%)
No answer	1 (0.9%)	6 (7.2%)	3 (2.8%)	3 (3.9%)	1 (14.3%)	7 (3.7%)
Total	109 (100.0%)	83 (100.0%)	108 (100.0%)	77 (100.0%)	7 (100.0%)	192 (100.0%)

**Table 2:** Reasons for choosing or avoiding self-medication practice.

Attitudes about Self-Medication	Study Program		Study Year *		Total *n* (%)
	Medicine *n* (%)	Pharmacy *n* (%)	First *n* (%)	Final *n* (%)	
Reasons for practicing self-medication					
Lack of free time	18 (20.9%)	8 (11.4%)	8 (10.3%)	17 (23.3%)	26 (16.6%)
Mild symptoms (perceived as unnecessary for a visit to a doctor)	55 (64.0%)	53 (75.7%)	55 (70.5%)	50 (68.5%)	108 (69.2%)
Distance to a doctor’s office	6 (7.0%)	1 (1.4%)	0 (0.0%)	6 (8.2%)	7 (4.48%)
Having a positive experience with the same medicine	36 (41.9%)	30 (42.9%)	34 (43.6%)	31 (42.5%)	66 (42.3%)
Other	1 (1.2%)	2 (2.9%)	0 (0.0%)	2 (2.7%)	1 (0.6%)
Reasons against practicing self-medication					
There was no need	18 (78.3%)	7 (53.8%)	21 (70.0%)	3 (75.0%)	26 (70.3%)
Due to safety concerns	5 (21.7%)	6 (46.2%)	9 (30.0%)	1 (25.0%)	11 (29.7%)

* one medical student and six pharmacy students did not answer the question regarding the study year; total for study year *n* = 185, total for whole sample *n* = 192.

**Table 3:** Sources of medicines used for self-medication and information regarding the choice and proper use.

Sources of Medicines and Information for Self-Medication	Study Program		*p*-Value	Study Year *		*p*-Value	Total *n* (%)
	Medicine *n* (%)	Pharmacy *n* (%)		First *n* (%)	Final *n* (%)		
Sources of medicines							
Pharmacy	78 (90.7%)	65 (92.9%)	*p* = 0.175	72 (92.3%)	68 (93.2%)	*p* = 0.385	143 (91.7%)
Relatives, friends, neighbors	8 (9.3%)	3 (4.3%)		4 (5.1%)	7 (9.6%)		11 (7.1%)
Traditional healer, homeopath	1 (1.2%)	1 (1.4%)		1 (1.3%)	1 (1.4%)		2 (1.3%)
Health food stores, herbal pharmacies	8 (9.3%)	6 (8.6%)		6 (7.7%)	7 (9.6%)		14 (9.0%)
Home pharmacies	43 (50.0%)	26 (37.1%)		32 (41.0%)	35 (47.9%)		69 (44.2%)
Sources of information on desired drug							
Mass media (TV, radio)	1 (1.2%)	3 (4.3%)	*p* = 0.486	2 (2.6%)	2 (2.7%)	*p* < 0.01	4 (2.6%)
Internet	16 (18.6%)	9 (12.9%)		14 (17.9%)	9 (12.3%)		25 (16.0%)
Relatives, friends, neighbors	24 (27.9%)	14 (20.0%)		23 (29.5%)	14 (19.2%)		38 (24.4%)
Pharmacist	27 (31.4%)	31 (44.3%)		34 (43.6%)	23 (31.5%)		58 (37.2%)
Medical doctor (from previous visits)	46 (53.5%)	33 (47.1%)		43 (55.1%)	34 (46.6%)		79 (50.6%)
Professional literature (class books)	34 (41.9%)	20 (28.6%)		10 (12.8%)	43 (58.9%)		54 (34.6%)

* one medical student and six pharmacy students did not answer the question regarding the study year; total for study year *n* = 185, total for whole sample *n* = 192.

**Table 4:** Prevalence of self-medication practice with herbal medicines in combination with conventional drugs and students’ view about their safety.

Claims about Conventional and Herbal Medication	Study Program		*p*-Value	Study Year *		*p*-Value	Total *n* (%)
	Medicine *n* (%)	Pharmacy *n* (%)		First *n* (%)	Final *n* (%)		
Concomitant use of conventional drugs and herbal preparations (in previous 12 months)							
Yes	44 (40.4%)	29 (34.9%)	*p* = 0.200	38 (35.2%)	32 (41.6%)	*p* = 0.669	73 (38.0%)
No	41 (37.6%)	41 (49.4%)		39 (36.1%)	41 (53.3%)		82 (42.7%)
No answer	24 (22.0%)	13 (15.7%)		31 (28.7%)	4 (5.2%)		37 (19.3%)
Reasons for their concomitant use of conventional drugs and herbal preparations **							
They’re used for different indications	1 (2.3%)	6 (20.7%)	*p* = 0.004	3 (7.9%)	3 (9.4%)	*p* = 0.364	7 (9.6%)
I think they act better in combination	12 (27.3%)	5 (17.2%)		7 (18.4%)	10 (31.3%)		17 (23.3%)
I do not think it is harmful	30 (68.2%)	13 (44.8%)		27 (71.1%)	14 (43.8%)		43 (58.9%)
Other	1 (2.3%)	1 (3.4%)		0 (0.0%)	2 (6.3%)		2 (2.7%)
Preference for one option to continue the therapy (if required)							
Conventional drug	71 (65.1%)	45 (54.2%)	*p* = 0.009	51 (47.2%)	62 (80.5%)	*p* = 0.045	116 (60.4%)
Herbal drug/preparation	15 (13.8%)	25 (30.1%)		27 (25.0%)	11 (14.3%)		40 (20.8%)
No answer	23 (21.1%)	13 (15.7%)		30 (27.8%)	4 (5.2%)		36 (18.8%)
Attitudes on the efficacy of herbal drugs							
Herbal drugs are more efficient	4 (3.7%)	6 (7.2%)	*p* = 0.45	8 (7.4%)	1 (1.3%)	*p* = 0.001	10 (5.2%)
Conventional drugs are more efficient	39 (35.8%)	32 (38.6%)		25 (23.2%)	44 (57.1%)		71 (37.0%)
They are equally efficient	14 (12.8%)	15 (18.1%)		15 (13.9%)	14 (18.2%)		29 (15.1%)
Not sure	29 (26.6%)	17 (20.5%)		30 (27.8%)	14 (18.2%)		46 (24.0%)
No answer	23 (21.1%)	13 (15.7%)		30 (27.8%)	4 (5.2%)		36 (18.8%)
Attitudes on the safety of herbal drugs							
Herbal drugs have less adverse effects	35 (31.8%)	30 (36.6%)	*p* = 0.001	41 (37.6%)	22 (28.9%)	*p* = 0.004	66 (34.4%)
Conventional drugs have less adverse effects	15 (13.6%)	17 (20.7%)		9 (8.3%)	22 (28.9%)		32 (16.7%)
They are equally safe	10 (10.0%)	15 (18.3%)		8 (7.3%)	17 (22.4%)		25 (13.0%)
Not sure	26 (23.6%)	7 (8.5%)		20 (18.3%)	12 (15.9%)		33 (17.2%)
No answer	23 (20.9%)	13 (15.9%)		31 (28.4%)	3 (3.9%)		36 (18.8%)
Did the pharmacist warn you about the risks of concomitant use of conventional and herbal drugs? ***							
Yes	9 (37.5%)	12 (60.0%)	*p* = 0.839	12 (48.0%)	9 (52.9%)	*p* = 0.734	21 (47.7%)
No	15 (62.5%)	8 (40.0%)		13 (52.0%)	8 (47.1%)		23 (52.3%)

* one medical student and six pharmacy students did not answer the question regarding the study year; total for study year *n* = 185, total for whole sample *n* = 192; ** answers shown for students who reported concomitant use of herbal and conventional drugs; *** answers shown for students who reported concomitant use of herbal and conventional drugs which they obtained at a pharmacy.

**Table 5:** Poisson regression model with robust variance using self-medication as a dependent variable.

Independent Variables	Univariate		Multivariate	
	PR (95% CI)	*p*-Value	PR (95% CI)	*p*-Value
Gender (female/male)	0.94 (0.79–1.11)	0.465		
Study program (pharmacy/medicine)	1.07 (0.94–1.22)	0.330		
Study year (final/first)	1.31 (1.16–1.49)	<0.001	1.28 (1.13–1.44)	<0.001
Housing conditions Living with parents In leased apartment/room In a student dormitory	reference 1.23 (1.04–1.45) 1.27 (1.04–1.56)	0.017 0.021	reference 1.25 (1.06–1.47) 1.33 (1.09–1.64)	0.008 0.006
Cigarette consumption	1.25 (1.13–1.38)	<0.001	1.20 (1.08–1.33)	0.001
Alcohol consumption	1.26 (1.17–1.36)	<0.001		
Chosen general practitioner	1.02 (0.87–1.20)	0.810		
Chronic illness	1.11 (0.94–1.32)	0.217		
Taking medication for chronic illnesses	1.04 (0.82–1.33)	0.754		

## Data Availability

All data are available by reasonable request.

## Supplementary Materials

The following supporting information can be downloaded at: https://www.mdpi.com/article/10.3390/ijerph19031193/s1, Supplementary File S1: The questionnaire: Self-Medication Perceptions and Practice.
