# Supplementary material for: Self-Medication Perceptions and Practice of Medical and Pharmacy Students in Serbia
Source: Int J Environ Res Public Health. 2022 Jan 21;19(3):1193. doi: 10.3390/ijerph19031193 (PMC8834465; doi:10.3390/ijerph19031193)
Supplement: Supplementary file 1 [file ijerph-19-01193-s001.zip › ijerph-1542956-supplementary.pdf]

Supplementary File S1. The questionnaire: Self-Medication Perceptions and Practice

PART I: Self-medication behavior

1. **Have you opted for self-medication, i.e. did you use any medication (including herbal and traditional products) for the illness and/or symptoms you identified, without consulting medical doctor, in previous 12 months?**
   - ☐ Yes
   - ☐ No

*If you answered **YES**, please go to question No. **3**.*

2. **What were your reasons for not using self-medication in previous 12 months? (check more than one if applicable)**
   - ☐ There was no need
   - ☐ Due to safety concerns, I thought it's safer to visit a physician
   - ☐ Not sure
   - ☐ Other (specify): ____

Please go to Part II, question No. 1.

3. **What were your reasons for using self-medication in previous 12 months? (check more than one if applicable)**
   - ☐ Lack of free time
   - ☐ I considered symptoms to be mild and not neccessary to see a doctor
   - ☐ Distance of doctor's office
   - ☐ I've already had positive experience with the medicine I used
   - ☐ Other (specify): ____

4. **For which symptoms/illnesses have you used medication on your own, i.e. without consulting medical doctor? (check more than one if applicable)**
   - ☐ Cold/flu (cough, runny nose, etc.)
   - ☐ Nausea, diarrhea, constipation, heartburn
   - ☐ Pain (headache, menstrual cramps, etc.)
   - ☐ Dermatological disorders (allergies, acne, etc.)
   - ☐ Elevated blood pressure, elevated cholesterol levels, peripheral vascular disease
   - ☐ Insomnia, depression, psychological disturbances
   - ☐ To improve general wellbeing and to boost immunity
   - ☐ Other (specify): ____

5. **Which medication classes have you used on your own, i.e. without consulting medical doctor?* (check more than one if applicable)**
   - ☐ Teas and other herbal preparations for therapeutic purpose
   - ☐ Vitamins and minerals
   - ☐ Homeopathic medicines
   - ☐ Dietary supplements
   - ☐ Conventional (standard) drugs
   - ☐ If not sure, specify the name: ______________________________________

*If you are not sure about the appropriate class of some product, type its name in the first field ('medication name') in the following link of Medicines and Medical Devices Agency of Serbia: https://www.alims.gov.rs/ciril/lekovi/pretrazivanje-humanih-lekova/

6. **How were you informed about the medicine you used in self-medication? (check more than one if applicable)**
   - ☐ Via mass-media (TV, radio)
   - ☐ Via Internet
   - ☐ By relatives, friends, neighbors
   - ☐ By a pharmacist
   - ☐ By a medical doctor (from previous visits for he same symptoms/illness)
   - ☐ From professional literature (classboks, handbooks)

7. **How did you obtain the medication you needed? (check more than one if applicable)**
   - ☐ In a pharmacy
   - ☐ From relatives, friends, neighbors
   - ☐ Purchasing on Internet
   - ☐ From healer, homeopath
   - ☐ In health food stores, herbal pharmacies
   - ☐ From households (drug leftovers from previous treatments)

8. **Have you used herbal drugs/preparations in combination with conventional drugs in previous 12 months?**
   - ☐ Yes
   - ☐ No

*If you answered **NO**, please go to question No. **12**.*

9. **What were the reasons for concomitant use of conventional drugs and herbal preparations?**
   - ☐ I didn't use them for the same symptom/illness
   - ☐ I think they act better in combination
   - ☐ I think it cannot be harmful, just useful
   - ☐ Other (specify): ____

10. **If I had to choose only one treatment option, I would continue my therapy with:**
    - ☐ Conventional drug
    - ☐ Herbal drug/preparation

11. **What do you think about the efficacy of herbal drugs?**
    - ☐ Herbal drugs are more efficient
    - ☐ Conventional drugs are more efficient
    - ☐ They are equally efficient
    - ☐ Not sure

12. **What do you think about the safety of herbal drugs?**
    - ☐ Herbal drugs have less adverse effects
    - ☐ Conventional drugs have less adverse effects
    - ☐ They are equally safe
    - ☐ Not sure

13. **Did a pharmacist warn you about the risks of concomitant use of conventional and herbal drugs?**
    - ☐ Yes
    - ☐ No
    - ☐ I didn't buy herbal drug in pharmacy

Part II: General information

1. **Age (years): _________________**

2. **Gender: A. Male B. Female**

3. **Study program:**
   - ☐ Integrated studies of Medicine
   - ☐ Integrated studies of Pharmacy

4. **Study year: _________________**

5. **Who do you live with during the semesters, i.e. in period you attend classes at the Faculty?**
   - ☐ With parents
   - ☐ In a student dormitory (alone or with roommates)
   - ☐ In a leased apartment/room (alone or with roommates)
   - ☐ Other (specify): _________________

6. **Do you consume cigarettes on a daily basis?**
   - ☐ Yes
   - ☐ No

7. **Do you consume alcohol more than 3 times per week?**
   - ☐ Yes
   - ☐ No

8. **Do you have your chosen general practitioner?**
   - ☐ Yes
   - ☐ No

9. **Do you have any chronic disease? Chronic diseases are persistent health conditions lasting longer than 3 months and therefore requiring long-term treatment, such as cardiovascular diseases (like heart attacks and stroke), cancers, chronic respiratory diseases (such as chronic obstructed pulmonary disease and asthma) and diabetes.**
   - ☐ Yes
   - ☐ No

10. **If you answere YES in question No. 9, do you take medications prescribed by medical doctor for that chronic disease?**
    - ☐ Yes
    - ☐ No
